# Removal of hematoma due to massive hemoptysis after transbronchial biopsy: a case report

**DOI:** 10.1186/s40792-021-01323-3

**Published:** 2021-11-03

**Authors:** Harushi Ueno, Hideki Tsubouchi, Keita Nakanishi, Tomoshi Sugiyama, Yuka Kadomatsu, Masaki Goto, Naoki Ozeki, Shota Nakamura, Takayuki Fukui, Toyofumi Fengshi Chen-Yoshikawa

**Affiliations:** Department of Thoracic Surgery, Nagoya University Graduate School of Medicine, 65 Tsurumai-cho, Showa-ku, Nagoya, 466-8550 Japan

**Keywords:** Transbronchial biopsy, Hemoptysis, Sutured shut

## Abstract

**Background:**

Massive hemoptysis is a life-threatening complication after transbronchial biopsy (TBB). Reports on massive hemoptysis occurring several days after TBB are scarce.

**Case presentation:**

A 62-year-old man presented with massive hemoptysis and was admitted to hospital as an emergency on the eighth day after TBB. On the 12th day after TBB, computed tomography showed complete atelectasis of the right middle and lower lobes. The patient underwent emergent right upper lobectomy. The right upper lobe bronchus was separated with a scalpel, the hematoma was pulled out with forceps, and the bronchus subsequently sutured shut. The patient was discharged from the hospital uneventfully.

**Conclusions:**

We experienced a case of massive hemoptysis on the eighth day after TBB, which required emergency surgery due to persistent bleeding into the airway and airway obstruction during follow-up. Postoperative pneumonia and atelectasis could be prevented by manual removal of the residual hematoma.

## Background

Bleeding is an associated complication after thoracic transbronchial biopsy (TBB), which is sometimes life-threatening. Massive hemoptysis, which is difficult to stop with compression hemostasis or hemostatic sprays after biopsy, occurs in 0.06% of all cases and may require broncho-embolization or bronchial artery embolization [1]. For uncontrolled massive hemoptysis that is difficult to stop with these techniques, surgical treatment including lobectomy is required [2]. Hemoptysis most often occurs immediately after TBB, and reports of massive hemoptysis after about a week are rare.

## Case presentation

**Fig. 1:**
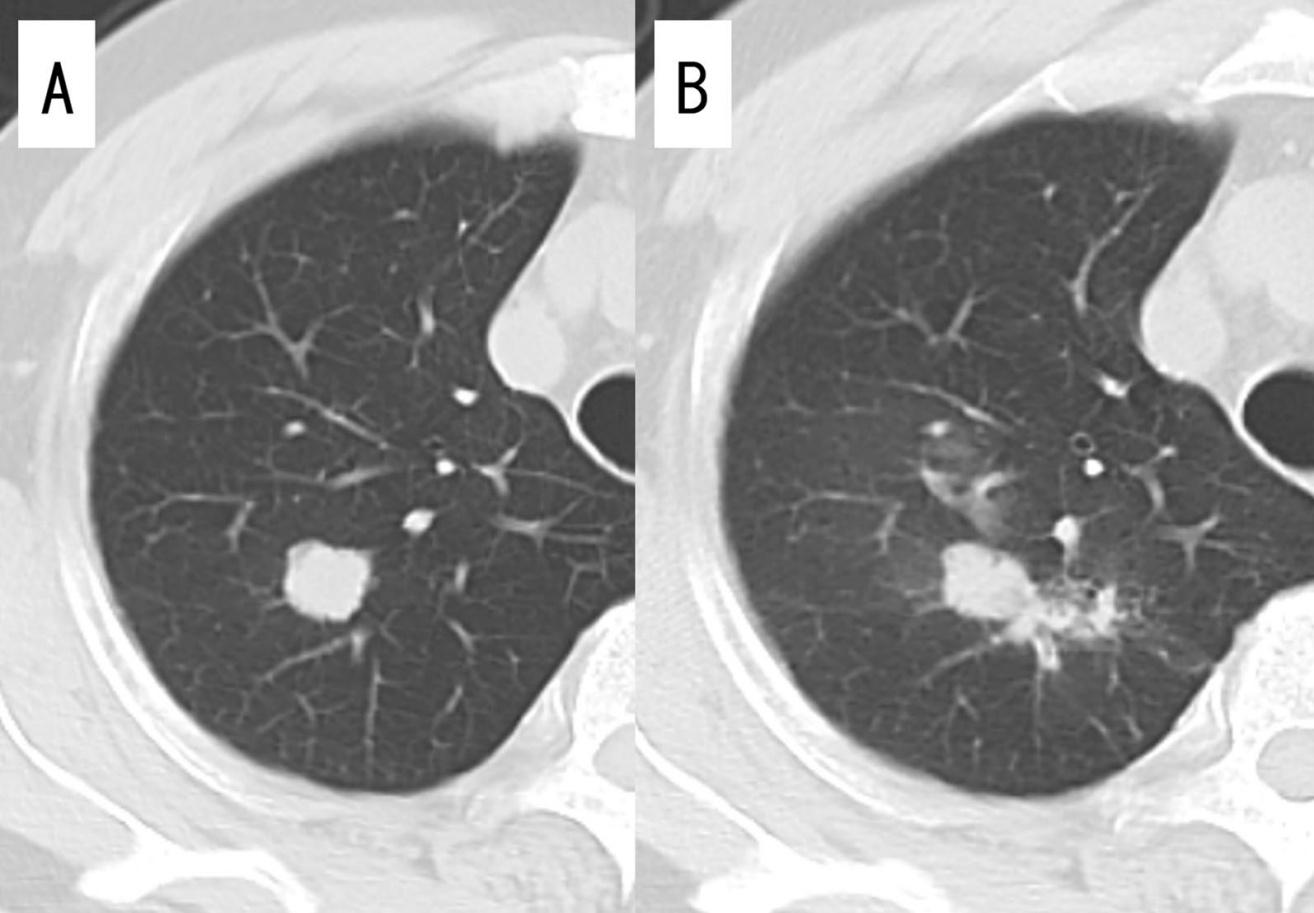
Computed tomography (CT) images. **A** On initial examination, CT showing a nodule in the right upper lobe and **B** after massive hemoptysis on the eighth day after bronchoscopy. Scattered shadows, presumably blood, are confined to the upper lobe

**Fig. 2:**
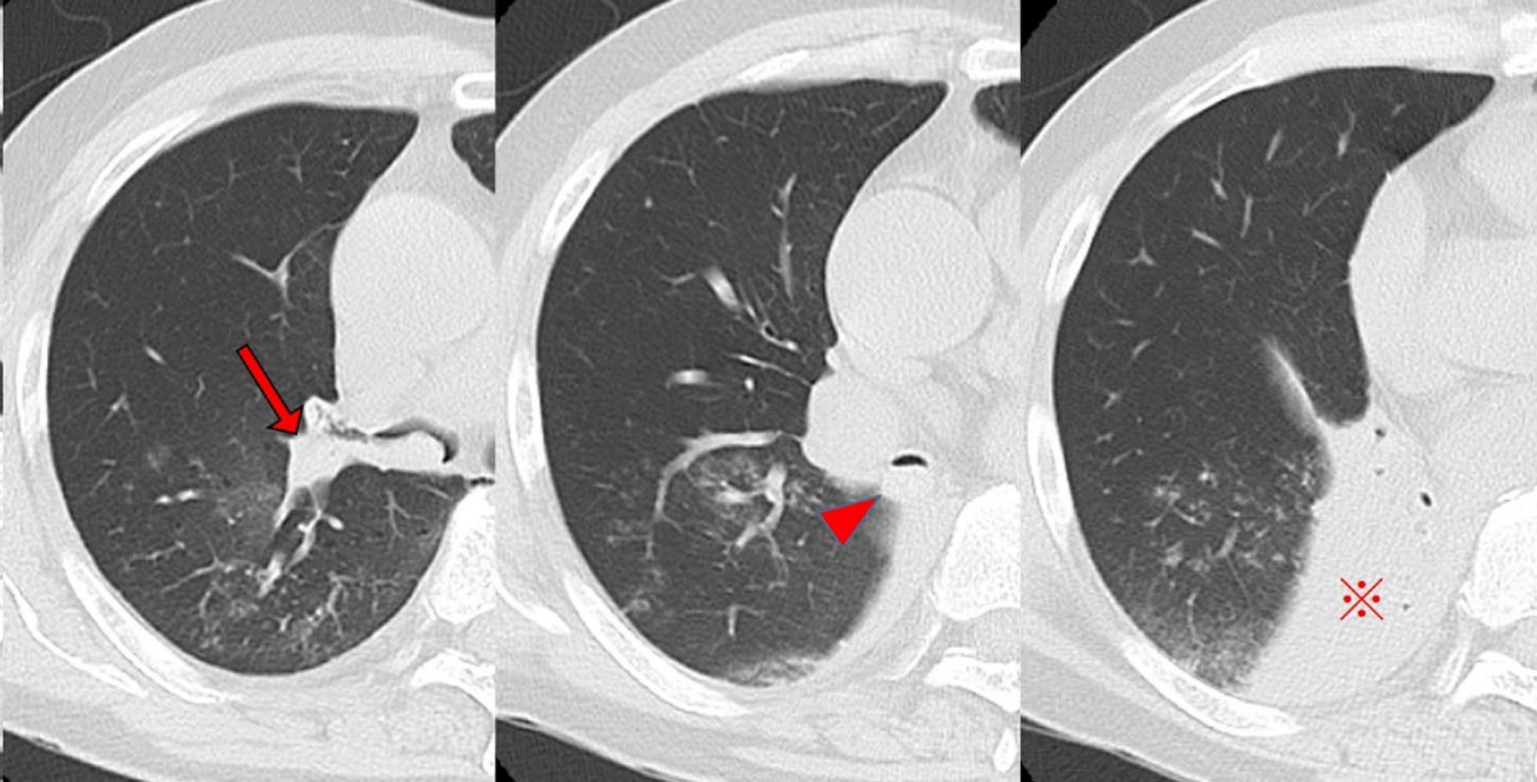
Intratracheal obstruction due to hematoma from the upper lobe bronchus (arrow) to the middle bronchial trunk (arrowhead). The right middle and lower lobes (※) were completely atelectatic

**Fig. 3:**
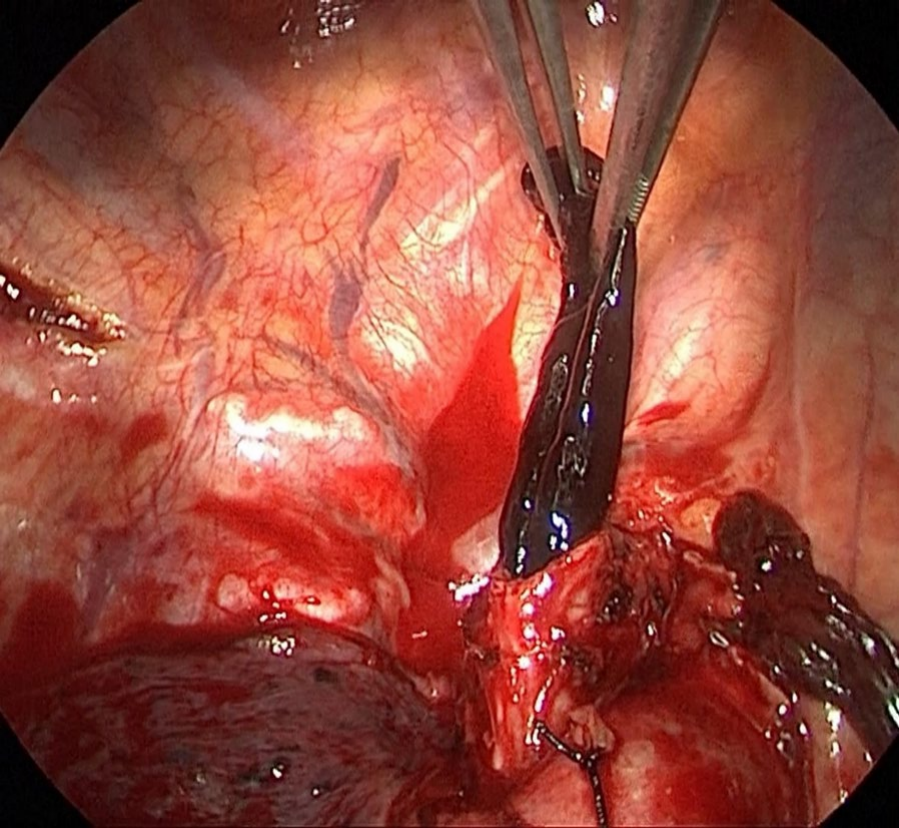
The hematoma in the airway was carefully pulled out with forceps from the end of the bronchus that had been separated with a scalpel

The patient was a 62-year-old man who presented with a nodule in the right upper lobe on X-ray during a medical examination. He had no significant medical history or medication, and blood chemistry findings were normal, including tumor markers and coagulation tests. Chest computed tomography (CT) showed a nodule with a maximum diameter of 20 mm in the right S1 region without enlargement of the lymph nodes (Fig. 1A). Contrast-enhanced magnetic resonance imaging of the head showed no brain metastasis. TBB was performed, and small-cell carcinoma was diagnosed. A small amount of hemoptysis was observed after TBB, but the symptoms disappeared the next day, and the patient was discharged from the hospital upon unremarkable findings on X-ray. Since the patient had small-cell lung cancer, positron emission tomography (PET)–CT was scheduled to assess for distant metastasis. On the eighth day after TBB, the patient was admitted to the hospital as an emergency due to 200–300 mL of hemoptysis. Contrast-enhanced CT of the chest showed scattered shadows around the right upper lobe nodule suggestive of hemorrhage (Fig. 1B). Since hemoptysis was reduced after admission, we continued conservative treatment with hemostatic agents and bronchial artery embolization was not performed. On the 12th day after TBB, SpO_2_ decreased to 92%, and chest CT showed occlusion of the upper lobe bronchus and the right middle bronchial trunk with hematoma, resulting in complete atelectasis of the right middle and lower lobes (Fig. 2). Emergency thoracotomy of the right upper lobe lobectomy (RUL) and lymph node dissection 2a-2 (ND2a-2) was performed. The operation was performed with differential lung ventilation through a double-lumen tube. The hematoma was occluding the right main bronchus and more centrally located than on the preoperative CT image. The hematoma in the airway was too viscous to be aspirated with suction pressure from the intubation tube. All lobes of the right lung were inflated, and achieving deflation was complicated by the obstruction of the right main bronchus. The pulmonary artery was identified from the interlobar space, and the ascending pulmonary artery was ligated. The minor fissure was dissected first, followed by the superior pulmonary vein and truncus pulmonary artery in this order. The right upper lobe was removed by dissecting the right upper bronchus with a scalpel, and the hematoma was carefully pulled out from the dissected end of the bronchus with forceps (Fig. 3). A suction tube was inserted into the middle and lower lobe bronchus to aspirate the remaining hematoma. The upper lobe bronchus was closed with six stitches using the Sweet method with 4–0 Prolene sutures. The pathological diagnosis was a large-cell neuroendocrine carcinoma with a maximum diameter of 20 mm and pT1bN0M0 Stage I A2. Chest CT on postoperative day 7 showed no atelectasis and good lung expansion. The patient was discharged uneventfully on postoperative day 10.

## Discussion

Bleeding after TBB led to the formation of a hematoma in the right airway, which caused ventilation failure in the right lung and required surgery for treatment. Although histopathological diagnosis by TBB was small-cell carcinoma, and PET–CT had not been performed, RUL + ND2a-2 was performed as it was a limited disease with an indication for surgery. Despite the preoperative CT finding of complete atelectasis in the right middle and lower lobes, at the time of surgery, all right lobes were fully expanded and could not be deflated due to central airway obstruction. Positive pressure ventilation during anesthesia induction may have caused the hematoma in the airway to act as a check valve and inflate the right lung. Intraoperatively, in order to remove the hematoma manually from the airway, the blood vessels were dissected first and then the airway. Suction from the dissected bronchus was used to aspirate as much of the residual hematoma as possible to ensure adequate expansion of the remaining lung. Differential lung ventilation is essential for lobectomy with airway hemorrhage, but the narrow lumen of the double-lumen tube made it difficult to aspirate the hematoma with a bronchoscope. Conversely, a single-lumen tube and blocker poses a risk of aspiration of blood into the left lung. In such cases, the method we have used is effective. Although the aspiration and removal of the hematoma through an incision in the posterior bronchial wall could have resulted in a collapsed lung and made the operation easier, the removal of the hematoma could have allowed fresh bleeding to enter the other lung lobe. We therefore decided to remove the hematoma after the removal of the upper lobe bronchus with a scalpel.

## Conclusions

We experienced a case of massive hemoptysis on the eighth day after TBB, which required emergency surgery due to persistent bleeding into the airway and airway obstruction during follow-up. Even after the patient stopped bleeding, the chest CT showed an enlarged hemorrhagic shadow, which required emergency surgery. Although the lobectomy was difficult because the lung did not collapse during surgery due to central airway obstruction, postoperative pneumonia and atelectasis could be prevented by manual removal of the residual hematoma.

## Data Availability

The data that support the findings of this study are available from the corresponding author upon reasonable request.

## Abbreviations

TBB: Transbronchial biopsy
CT: Chest computed tomography
PET: Positron emission tomography
RUL: Right upper lobe lobectomy
